# Green Synthesis of N-Doped Carbon Quantum Dots from Chitin Nanohydrogels for Highly Sensitive Fe^3+^ Detection

**DOI:** 10.3390/gels12040271

**Published:** 2026-03-25

**Authors:** Tianji Li, Delong Dai, Luohui Wang, Minghui Zhao, Lianfeng Shen, Youming Dong, Fei Xiao, Cheng Li, Jianwei Zhang

**Affiliations:** 1 College of Forestry, Henan Agricultural University, Zhengzhou 450046, China; 2 School of Earth and Space Science and Technology, Wuhan University, Wuhan 430072, China; 3 College of Materials Science and Engineering, Nanjing Forestry University, Nanjing 210037, China; 4 Hunan Academy of Forestry Sciences, Changsha 410018, China; 5 College of Pharmacy, Changsha Medical University, Changsha 410219, China

**Keywords:** chitin, nano-hydrogel, nitrogen-doped, carbon quantum dots, detection

## Abstract

In order to achieve rapid and qualitative detection of soluble heavy metal ions, nitrogen-doped fluorescent carbon quantum dots (N-CQDs) were synthesized using chitin extracted from shrimp and crab shells as the carbon source. The structural, morphological, and optical properties of the synthesized N-CQDs were systematically characterized using transmission electron microscopy (TEM), field emission scanning electron microscopy (FE-SEM), Fourier transform infrared spectroscopy (FTIR), Raman, X-ray photoelectron spectroscopies (XPS), ultraviolet-visible (UV-Vis) absorption spectroscopy and fluorescence spectroscopy. The resulting N-CQDs exhibited a carbonization yield of 54.46% and a fluorescence quantum yield of 34.33%. Their morphology, structure and optical properties were thoroughly characterized using a range of analytical techniques. The synthesized N-CQDs exhibited excellent fluorescence properties, and remarkable stability. When applied for metal ion detection, the N-CQDs displayed a distinct and selective fluorescence quenching response exclusively toward Fe^3+^ ions. The detection limit for Fe^3+^ at room temperature was 4.04 μmol/L. Furthermore, due to the inherent nitrogen present in the acetyl amino groups of chitin, nitrogen doping was achieved without the need for external dopants during the hydrothermal synthesis process. Owing to their high stability, low cost and low toxicity, the N-CQDs synthesized in this study provide a promising fluorescence sensing platform with excellent selectivity for Fe^3+^ detection, achieved through precise control of surface functional groups.

## 1. Introduction

In the past decade, increasing attention has been directed toward developing rapid, simple and real-time analytical techniques for detecting chemically and biologically important compounds, including drugs, biomolecules and metal ions. Among these, the highly sensitive detection of heavy metal ions, transition metal ions and organic pollutants has become particularly significant due to their environmental and health impacts.

Iron (Fe^3+^) in particular is of significant interest because it is both essential and potentially toxic. Fe^3+^ is widely found in natural and environmental water bodies and plays a vital role in biological systems through complexation with various regulatory proteins. However, excessive or prolonged exposure to Fe^3+^ can cause serious health issues, including cirrhosis, osteoporosis, and Alzheimer’s disease [1]. According to China’s current Drinking Water Sanitation Standard, the permissible limit for Fe^3+^ in drinking water is 0.3 mg/L [2]. Therefore, the development of analytical methods capable of selective Fe^3+^ detection in environmental samples is of great importance. Although a variety of traditional analytical methods—such as atomic absorption spectrometry, inductively coupled plasma mass spectrometry, and colorimetry—are available for Fe^3+^ detection, these techniques are often constrained by high cost, complex sample preparation, and unsuitability for real-time monitoring [3]. Recently, fluorescent materials, including semiconductor quantum dots and organic dyes, have emerged as promising candidates for metal ion sensing. Nevertheless, their applications remain limited due to complex synthesis procedures, strict detection conditions, and high production costs [4,5]. Therefore, there is a growing need to develop rapid, simple, low-cost and environmentally friendly methods for synthesizing biocompatible fluorescent nanomaterials that can offer high sensitivity for metal ion detection.

Among the various fluorescent nanomaterials discovered, carbon quantum dots (CQDs) have attracted significant attention due to their unique physical, chemical and optical properties [6,7,8]. CQDs are a novel class of zero-dimensional, spherical nanomaterials [9] with particle sizes typically below 10 nm, composed primarily of carbon (C), hydrogen (H) and oxygen (O). They are widely regarded as environmentally friendly carbon-based nanomaterials [10]. Structurally, the core of CQDs consists of sp^2^ and sp^3^ hybridized carbon or amorphous carbon, while their surface is enriched with abundant hydroxyl, carboxyl, amino and other functional groups. These structural characteristics endow CQDs with excellent water solubility, chemical and optical stability, and biocompatibility [11,12]. In addition, CQDs exhibit remarkable optical properties, including strong photoluminescence, high light stability, and photobleaching resistance, making them promising candidates to replace traditional semiconductor quantum dots and organic dyes [13]. Owing to these advantages, CQDs have found broad applications in environmental and ion detection [14], biosensing [15], bioimaging [16], cell labeling [17], and drug delivery [18]. The origin of CQD research can be traced back to 1985, when scientists in the United Kingdom and the United States first synthesized a hollow carbon-based molecule known as fullerene [19]. Later, in 2004, Xu et al. [20] discovered luminescent carbon nanoparticles—now known as CQDs—while synthesizing carbon nanotubes via the arc-discharge method, revealing their superior optical properties. In recent years, CQDs have emerged as promising fluorescent probes for Fe^3+^ detection owing to their excellent photostability, biocompatibility, and tunable optical properties. Mandal et al. synthesized GQDs via pyrolysis of citric acid, achieving a detection limit of 40 ± 2 nM for Fe^3+^ through Förster resonance energy transfer (FRET) mechanism [21]. Li et al. reported nitrogen-doped and amino acid-functionalized GQDs from glycine thermolysis, demonstrating selective Fe^3+^ sensing with a detection limit of 0.1 μM over the range of 0.5 μM to 0.5 mM [22]. Ju et al. developed nitrogen-doped GQDs through hydrothermal treatment with hydrazine, showing enhanced fluorescence (QY = 23.3%) and high sensitivity toward Fe^3+^ detection with a detection limit of 90 nM in the range of 1–1945 μM [23].

Currently, various methods have been developed for the synthesis of CQDs, which can generally be categorized into “top-down” and “bottom-up” approaches. The top-down method involves breaking down larger carbon structures, such as graphene, into nanoscale carbon quantum dots through techniques such as arc discharge, chemical oxidation, electrochemical oxidation, and laser etching [24]. Although these methods enable the resulting CQDs to inherit certain morphological characteristics of the parent material, they typically require high energy consumption and involve harsh chemical reagents, such as strong acids or bases, which limit their environmental sustainability. In contrast, the bottom-up method synthesizes carbon quantum dots from smaller organic molecules or biomass-derived precursors such as sugar, lignin, and agricultural residues, typically under hydrothermal or microwave-assisted conditions [25]. Specific techniques include the template, ultrasonic, microwave and hydrothermal methods [26]. CQDs synthesized through this route can retain the molecular structure characteristics of the raw materials and possess controllable surface chemistry, topology, and microstructure [27]. However, most of these conventional synthesis routes still face challenges such as low yield, harsh reaction conditions, and the need for expensive surface passivating agents to enhance fluorescence properties and metal ion selectivity [28]. Therefore, developing a simple, economical and environmentally friendly method for synthesizing CQDs from waste-derived materials without the need for a surface passivator is both scientifically significant and environmentally beneficial.

Chitin, also known as chitosan in its deacetylated form, is the main component of marine shellfish shells. As a polysaccharide second only to cellulose in abundance, chitin is the most prevalent natural nitrogen-containing organic polymer apart from proteins and possesses excellent biocompatibility [29]. Consequently, nanomaterials derived from chitin and its derivatives have gained increasing attention due to their renewable nature and versatile functionality. In this study, chitin was dissolved in a NaOH–urea solvent system and homogenized via high-speed mixing to obtain chitin nanogels through a one-step synthesis without the need for chemical modification or crosslinking agents. The resulting nanogels exhibited particle sizes ranging from 20 to 30 nm with a yield approaching 100%. Subsequently, deacetylation and Tempo-catalyzed oxidation were employed to obtain positively charged amino-chitin nanohydrogels (DE-CNGs) and negatively charged carboxylated chitin nanohydrogels (CO-CNGs), respectively [30]. The electrostatic repulsion generated by surface charges improved the dispersion uniformity of the modified nanohydrogels compared with the unmodified chitin nanogels. In addition, since chitin inherently provides C and N sources, it enables the green synthesis of nitrogen-doped carbon quantum dots (N-CQDs) through a simple, environmentally friendly route without the addition of exogenous nitrogen dopants. This approach effectively addresses the low quantum yield typically associated with CQDs derived from natural materials, thereby offering a sustainable strategy for producing high-performance fluorescent nanomaterials.

In this study, nitrogen-doped carbon quantum dots (N-CQDs) were successfully synthesized through a simple and environmentally friendly method without the use of surface passivating agents or exogenous nitrogen sources. The structural and optical properties of the N-CQDs were comprehensively analyzed, revealing their excellent fluorescence characteristics and high selectivity toward Fe^3+^ ions. The effects of various surface functional groups on fluorescence intensity were also investigated to optimize the synthesis conditions, thereby improving the selectivity of the N-CQDs for Fe^3+^ detection. A quantitative linear relationship between the fluorescence response of carbon quantum dots and Fe^3+^ ion concentration was established. Our work offers several distinct advantages: (1) a significantly higher quantum yield (34.33%) compared to most reported CQD sensors (typically 15–30%), which is advantageous for both visual detection and sensitive instruments; (2) a much wider linear range (0–5000 μM) enabling detection across a broad concentration span suitable for various water samples; (3) a green, one-pot synthesis without additional nitrogen-doping agents or toxic chemicals; and (4) the utilization of renewable biomass waste (shrimp/crab shells) as a sustainable carbon source. This study offers a novel and environmentally friendly approach for the rapid determination of Fe^3+^ in water and provides new insights into the design and synthesis of N-CQDs for advanced sensing applications.

## 2. Results and Discussion

### 2.1. Characterization and Analysis of CNGs and N-CQDs

**Figure 1:**
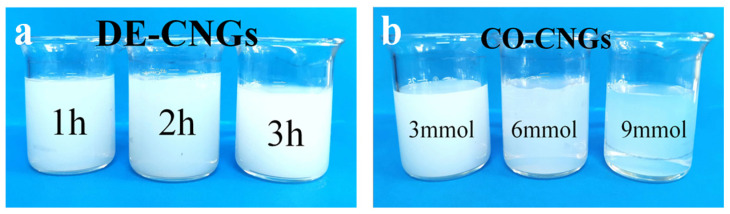
Photographs of (**a**) DE-CNGs obtained at different deacetylation times and (**b**) CO-CNGs prepared with different amounts of NaClO during TEMPO-catalyzed oxidation.

Deacetylation and Tempo-catalyzed oxidation of CNGs were successfully carried out to obtain amino-chitin nanogels (DE-CNGs) and carboxylated chitin nanogels (CO-CNGs), respectively. As shown in Figure 1, the morphology of DE-CNG showed no significant change with increasing deacetylation time, indicating that the structural integrity of the nanogels was maintained during the reaction. In contrast, as the amount of sodium hypochlorite increased during TEMPO-mediated oxidation, CO-CNGs exhibited improved dispersion stability and reduced aggregation, resulting in more transparent dispersions (Figure 1b). This enhanced colloidal stability can be attributed to the increased density of negatively charged carboxyl groups on the nanogel surface, which strengthens electrostatic repulsion between particles and prevents aggregation [31,32]. As shown in Table 1, the zeta potentials of DE-CNGs increased progressively from +27.2 mV to +34.3 mV with increasing deacetylation time (1–3 h), reflecting a higher density of protonated amino groups (–NH_3_^+^) generated by prolonged alkaline deacetylation. In contrast, CO-CNGs exhibited increasingly negative zeta potentials (−12.1 to −22.2 mV) as the NaClO dosage rose from 3 to 9 mmol, consistent with the progressive oxidation of surface hydroxyl groups to carboxylate groups (–COO–) via TEMPO-mediated oxidation. All modified nanogels displayed zeta potential magnitudes sufficient to maintain colloidal stability through electrostatic repulsion, which is further evidenced by the improved dispersion transparency of CO-CNGs observed in Figure 1b. These results confirm that both deacetylation time and NaClO dosage serve as effective parameters for tuning the surface charge density of chitin nanogels.

**Figure 2:**
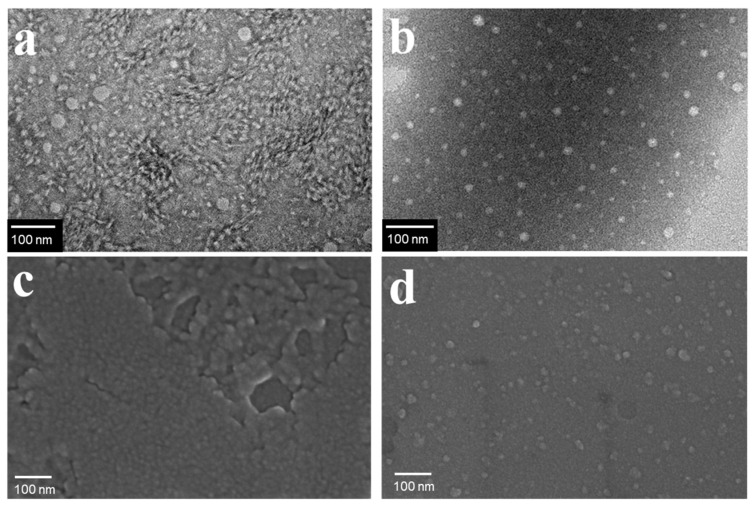
TEM images of (**a**) DE-CNGs and (**b**) CO-CNGs; SEM images of (**c**) DE-CNGs and (**d**) CO-CNGs; Scale bars = 100 nm. (**a**,**c**) DE-CNGs prepared by 2 h deacetylation treatment; (**b**,**d**) CO-CNGs prepared with 6 mmol NaClO oxidation. These conditions were selected as representative samples for morphological characterization.

The prepared CNGs were further characterized by TEM and FE-SEM, and the corresponding images are presented in Figure 2. As shown, DE-CNGs appeared predominantly spherical or elliptical with particle sizes ranging from 20~30 nm, whereas CO-CNGs exhibited more uniformly dispersed spherical particles with a slightly smaller average diameter of around 20 nm. Compared with the unmodified CNGs, both sur-face-modified nanogels demonstrated improved dispersion stability in aqueous media, which is mainly attributed to electrostatic repulsion from surface charges [33].

**Figure 3:**
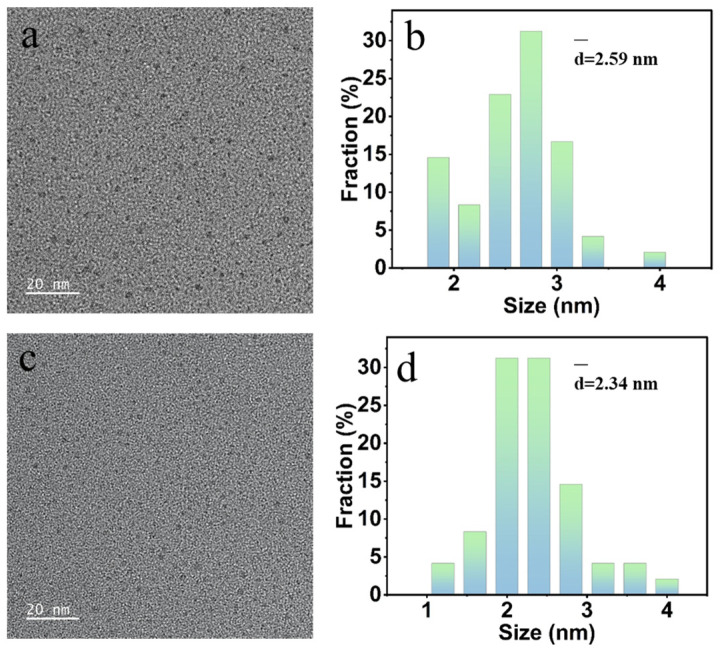
(**a**) TEM image and (**b**) particle size distribution of amino-N-CQDs synthesized from DE-CNGs after 2 h of deacetylation; (**c**) TEM image and (**d**) particle size distribution of carboxyl-N-CQDs synthesized from CO-CNGs with 6 mmol NaClO oxidation. Scale bars = 20 nm.

N-CQDs were synthesized through hydrothermal carbonization of DE-CNGs and CO-CNGs, respectively. As shown in Figure 3, both amino-N-CQDs and carboxyl-N-CQDs exhibited nearly spherical morphology with uniform dispersion and negligible aggregation. Statistical analysis of particle size based on 50 randomly selected individual particles revealed that both samples displayed narrow size distributions predominantly in the range of 1–4 nm, with a peak diameter of approximately 2 nm (Figure 3b,d). Notably, the particle size distributions of amino-N-CQDs and carboxyl-N-CQDs were highly similar, indicating that the type of surface functional group does not significantly alter the particle dimensions of the resulting N-CQDs. This finding rules out quantum size effects as the primary cause of the observed differences in fluorescence performance and Fe^3+^ detection sensitivity between the two samples and suggests that such differences should be attributed to the distinct surface chemistry of each modification type. These TEM results confirmed that N-CQDs were successfully synthesized via the described method and fulfill the morphological criteria of zero-dimensional quantum dot nanomaterials (particle size < 10 nm), in which quantum confinement effects are expected to contribute to the observed photoluminescence behavior.

**Figure 4:**
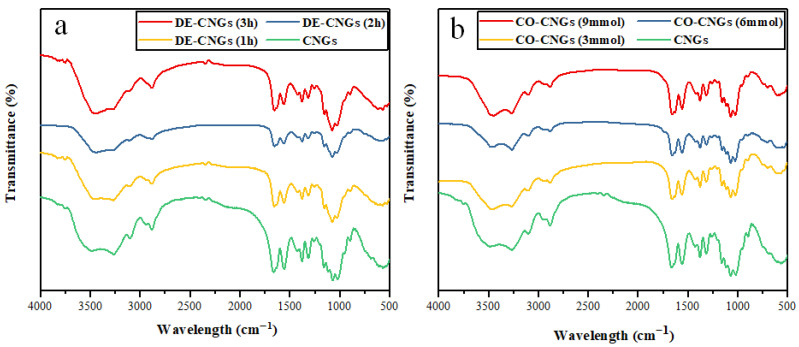
FTIR spectra of (**a**) DE-CNGs obtained after 1, 2 and 3 h of deacetylation and (**b**) CO-CNGs prepared with 3, 6 and 9 mmol of NaClO.

In order to assess potential structural changes in CNGs during surface modification, FTIR was conducted. The FTIR spectra of CNGs, DE-CNGs and CO-CNGs are shown in Figure 4. Compared with CNGs, DE-CNGs exhibited a characteristic absorption peak at 1600 cm^−1^, corresponding to the amino group [34], confirming the successful amination of nanogels. After carboxylation, the characteristic absorption peaks for C=O groups emerged at 1648 cm^−1^ and 1419 cm^−1^, indicating the conversion of hydroxyl groups at the C6 position into carboxyl groups [35]. Furthermore, the decreased intensity of the C–H stretching vibration at 2880 cm^−1^ suggests oxidation of hydroxyl groups to carboxyl groups [36,37]. These observations demonstrate that both amino and carboxyl modifications of CNGs were successfully achieved.

**Figure 5:**
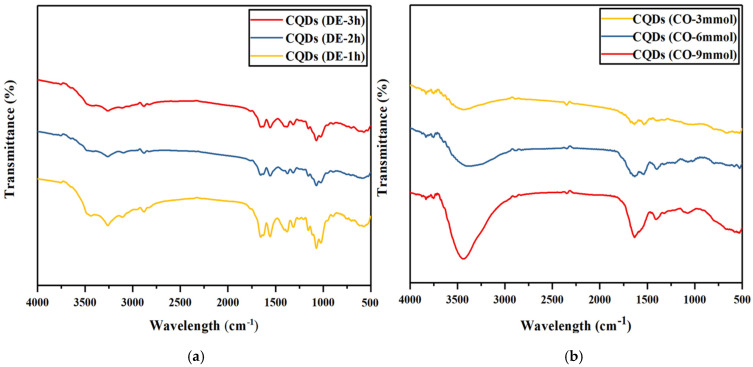
FTIR spectra of (**a**) amino-N-CQDs synthesized from DE-CNGs at varying deacetylation times (1, 2 and 3 h) and (**b**) carboxyl-N-CQDs synthesized from CO-CNGs at varying NaClO oxidation levels (3, 6 and 9 mmol, pH = 2).

To identify the surface functional groups of N-CQDs synthesized under different conditions, FTIR analysis was performed, and the resulting spectra are shown in Figure 5. After hydrothermal carbonization to N-CQDs, the FTIR spectra showed both similarities and differences compared to nanogel precursors. The similarities include the following points: broad band at 3200–3500 cm^−1^ (O–H, N–H) [38] remained, confirming surface hydrophilicity; peaks around 1600 cm^−1^ (aromatic C=C, N–H bending, COO–) were retained [39,40,41] and C–O stretching region (1000–1200 cm^−1^) persisted [42].

In contrast, there are some key differences indicating structural changes. Firstly, significantly reduced intensity of C-H stretching (2850–2950 cm^−1^), suggesting dehydrogenation and increased carbonization degree. Secondly, broadening and merging of peaks in the 1500–1700 cm^−1^ region, indicating formation of conjugated aromatic structures with overlapping C=C, C=N, and C=O vibrations. Lastly, relative increase in intensity of the 1000–1200 cm^−1^ region (C–O), suggesting concentration of oxygen-containing groups on N-CQD surfaces as aliphatic carbons are preferentially removed. In summary, FTIR data indicate that hydrothermal treatment partially converted chitin nanogels into more aromatic, carbonized structures while preserving important surface functional groups (O–H, N–H, COO–) that enable water solubility and metal ion binding [43].

**Figure 6:**
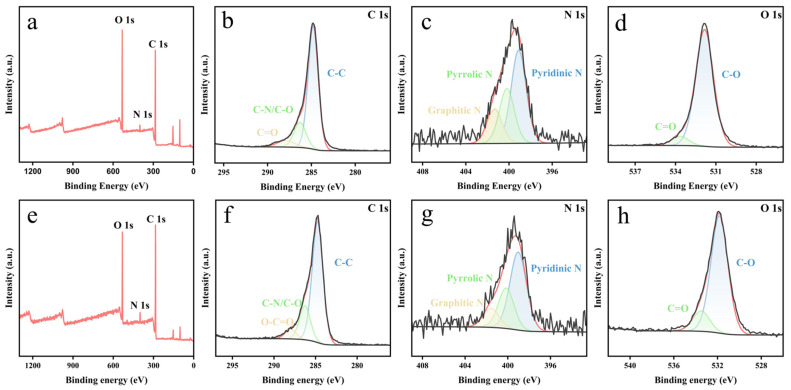
(**a**) Survey, (**b**) C 1s, (**c**) N 1s, (**d**) O 1s XPS spectra of amino-N-CQDs synthesized from DE-CNGs after 2 h of deacetylation; (**e**) survey, (**f**) C 1s, (**g**) N 1s, (**h**) O 1s XPS spectra of carboxyl-N-CQDs synthesized from CO-CNGs with 6 mmol NaClO oxidation.

The surface functional groups and elemental composition of the N-CQDs were analyzed using XPS. Figure 6 shows the wide scan spectra for amino-N-CQDs and carboxyl-N-CQDs. The expected carbon (C 1s), nitrogen (N 1s) and oxygen (O 1s) peaks are observed for both samples, confirming the successful incorporation of nitrogen into the carbon framework during hydrothermal carbonization of the chitin nanogel precursors.

In order to obtain more details on the types of bonds present, high-resolution scans were obtained and deconvoluted. For the C 1s spectra (Figure 6b,f), three peaks were observed for amino-N-CQDs at binding energies of ~284.8 eV, ~286 eV and ~288 eV, corresponding to C–C/C=C, C–N/C–O and C=O, respectively, showing the presence of these bonds in amino-N-CQDs. For carboxyl-N-CQDs, an additional peak at ~289 eV corresponding to O–C=O was observed, confirming the successful introduction of –COOH groups via TEMPO-mediated oxidation, which was absent in the amino-N-CQD spectrum.

For the high-resolution N 1s scans (Figure 6c,g), three peaks were observed at binding energies of ~398.5 eV, ~400.1 eV and ~401.3 eV for both samples, corresponding to pyridinic N, pyrrolic N and graphitic N, respectively, showing the presence of these nitrogen species in both N-CQD variants. For the O 1s spectra (Figure 6d,h), two peaks were observed at ~531 eV and ~533 eV for both samples, corresponding to C=O and C–O, respectively. The stronger O 1s signal intensity observed for carboxyl-N-CQDs is consistent with the higher surface density of oxygen-containing groups introduced by carboxylation.

**Figure 7:**
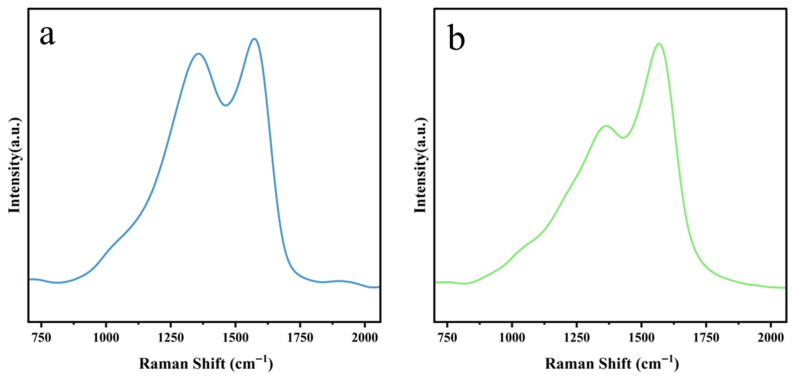
Raman spectra of (**a**) amino-N-CQDs synthesized from DE-CNGs after 2 h of deacetylation and (**b**) carboxyl-N-CQDs synthesized from CO-CNGs with 6 mmol NaClO oxidation.

Raman spectroscopy is instrumental in elucidating the structural characteristics associated with defects and graphitization in carbon-based nanomaterials. The Raman spectra of both N-CQDs exhibit two prominent peaks, characteristic of carbon dot materials (Figure 7). The first peak, located at approximately 1579.0 cm^−1^ (amino-N-CQDs) and 1573.7 cm^−1^ (carboxyl-N-CQDs), is identified as the G band. This peak signifies the presence of sp^2^ hybridization, characteristic of the in-plane stretching vibration of carbon atoms arranged in a hexagonal graphitic lattice, and serves as a hallmark of materials possessing a degree of regular crystalline order. Concomitantly, another peak emerges at 1358.5 cm^−1^ (amino-N-CQDs) and 1367.6 cm^−1^ (carboxyl-N-CQDs), denoted as the D band. This feature indicates the presence of disorder and structural defects within the carbon framework, often associated with sp^3^-hybridized carbon atoms, edge defects, and heteroatom substitution sites introduced during hydrothermal carbonization of the chitin nanogel precursors.

To quantify the relative extent of disorder and graphitization within the N-CQD carbon structure, the intensity ratio of the D band to the G band (*I_D_*/*I_G_*) was evaluated. For amino-N-CQDs and carboxyl-N-CQDs, the measured *I_D_*/*I_G_* ratios were 0.9435 and 0.6954, respectively. The higher *I_D_*/*I_G_* ratio observed for amino-N-CQDs indicates a greater density of structural defects introduced by amino group incorporation, which disrupts the regularity of the sp^2^ graphitic network and generates abundant sp^3^ edge defect sites. In contrast, the lower *I_D_*/*I_G_* ratio of carboxyl-N-CQDs reflects a relatively higher degree of graphitization, with the TEMPO-mediated carboxylation selectively targeting surface hydroxyl groups without substantially perturbing the sp^2^ carbon skeleton. These observations confirm the presence of surface hybrid functional groups on both N-CQD variants, consistent with the FTIR and XPS results. Notably, both *I_D_*/*I_G_* values fall within the range (0.69–1.01) reported for high-quality nitrogen-doped carbon dots, validating the structural integrity of the N-CQDs prepared in this study.

**Figure 8:**
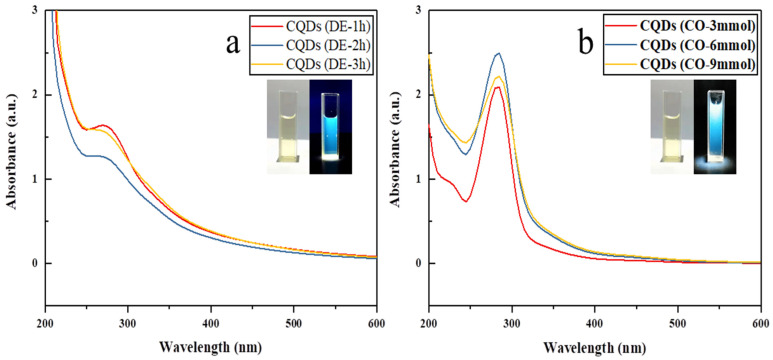
UV–Vis absorption spectra of (**a**) amino-N-CQDs synthesized from DE-CNGs after 1, 2 and 3 h of deacetylation and (**b**) carboxyl-N-CQDs synthesized from CO-CNGs with 3, 6 and 9 mmol NaClO oxidation. Inset: photographs of the N-CQDs solution under daylight (left) and UV light at 365 nm (right), showing its translucent brown color and bright blue fluorescence, respectively.

The optical properties of the synthesized N-CQDs were examined using UV-visible absorption spectroscopy under various preparation conditions. As shown in Figure 8, all N-CQDs exhibited a characteristic absorption peak at 270 nm, which is typically attributed to the π-π* transition of C=C bonds, suggesting the possible presence of sp^2^-hybridized aromatic domains within the N-CQD core [44]. This spectral feature may indicate the formation of conjugated carbon structures, which could contribute to the observed fluorescence emission through π-π* electronic transitions [45]. Additionally, the absorption band near 240 nm (Figure 6b) is attributed to the n-π* transition, which may be associated with amide bond formation between –NH_2_ and –C=O groups during CQDs synthesis.

**Figure 9:**
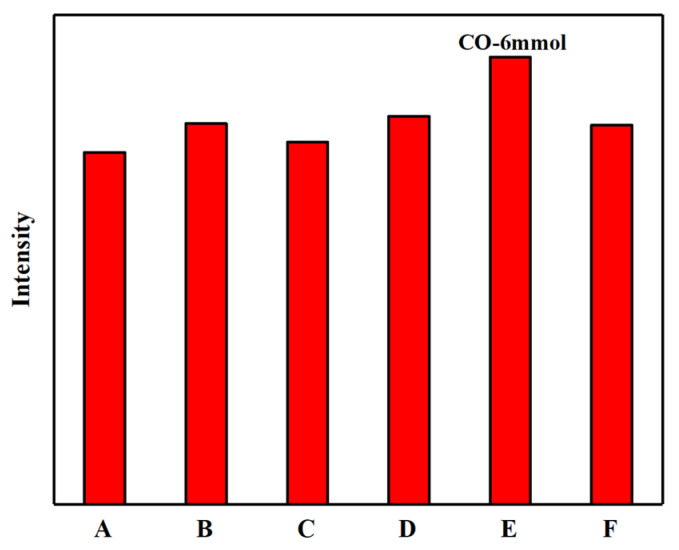
Fluorescence intensity of N-CQDs prepared under different conditions (A~F correspond to the following preparation conditions: DE–1 h, DE–2 h, DE–3 h, CO–3 mmol, CO–6 mmol, and CO–9 mmol).

In order to further investigate the photoluminescence properties of the synthesized N-CQDs, fluorescence intensities were measured for samples prepared under different modification conditions (see Figure 9). Among all samples, the N-CQDs derived from carboxylated chitin nanogels with 6 mmol NaClO (CO–6 mmol) exhibited the highest fluorescence intensity after heating. In both carboxyl-N-CQDs and amino-N-CQDs, the fluorescence intensity initially increased and then decreased with greater degrees of surface modification. Notably, the carboxyl-N-CQDs consistently exhibited stronger fluorescence than those amino-N-CQDs. This variation in fluorescence intensity can be attributed to differences in nanogel particle size, dispersion uniformity, and the quantity and type of surface functional groups [46,47]. Smaller nanogel particles provide a larger specific surface area, allowing for more efficient interaction with water and improved carbonization during the hydrothermal process. This, in turn, enhances the fluorescence of the resulting N-CQDs [48]. Therefore, the CO–6 mmol condition was selected for subsequent experiments due to its superior luminescent performance.

**Figure 10:**
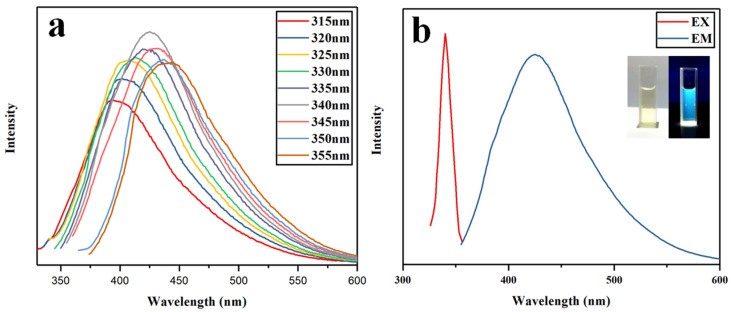
(**a**) Fluorescence emission spectra of N-CQDs at different excitation wavelengths and (**b**) optimal fluorescence excitation and emission spectra of N-CQDs. Inset: On the left side of the picture are N-CQDs under visible light, and on the right side of the picture are N-CQDs under the irradiation of 425 nm ultraviolet light.

Figure 10a shows the fluorescence spectra of N-CQDs (CO–6 mmol) under different excitation wavelengths. The spectra revealed that N-CQDs exhibited a strong excitation wavelength dependence. As the excitation wavelength increased, the emission peak redshifted by about 60 nm—a typical feature of chitosan-based quantum dots [49]. This redshift can be attributed to the non-uniform particle size distribution of N-CQDs, which influences light absorption and emission behavior, as well as to the presence of various surface defect structures introduced by oxygen-containing carboxyl groups or nitrogen dopants. When the excitation wavelength increased from 315 nm to 355 nm, the fluorescence intensity initially increased and then decreased, reaching its maximum at an excitation wavelength of 340 nm with an emission peak at 425 nm. Figure 10b displays the optimal excitation and emission spectra of N-CQDs, along with their fluorescence behavior under natural and ultraviolet illumination. The fluorescence quantum yield (QY) of the prepared N-CQDs was also determined using quinine sulfate solution (QY = 54%) as a reference. The absorbances of the quinine sulfate and N-CQD solutions (both at 10 mg/L) were measured as 0.27 and 0.13, respectively, yielding a calculated fluorescence quantum yield of 34.33% for the N-CQDs.

**Figure 11:**
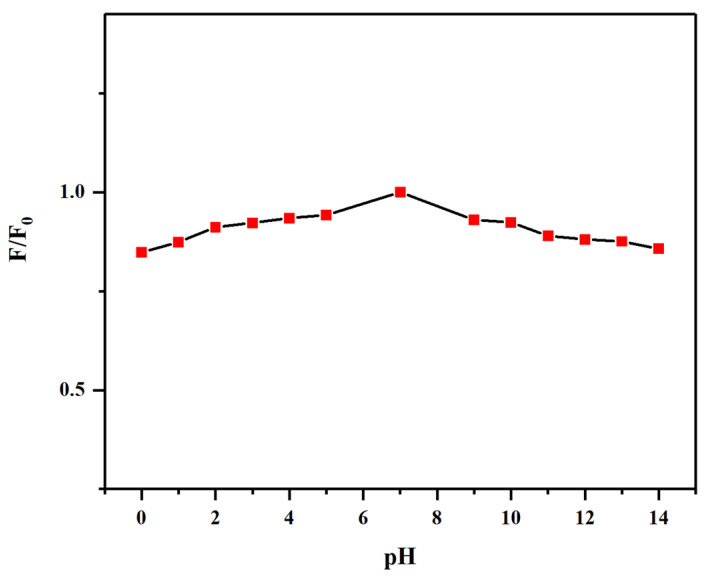
Effect of pH on fluorescence intensity of N-CQDs.

Figure 11 shows the fluorescence intensity of N-CQDs at an excitation wavelength of 340 nm under different pH conditions. As observed, N-CQDs exhibited the highest fluorescence intensity in a neutral environment (pH ≈ 7). With increasing acidity and alkalinity, the fluorescence intensity gradually decreased; however, the overall change remained minimal. This indicates that N-CQDs were relatively insensitive to changes in pH and maintained good fluorescence stability across a wide pH range. Such stability demonstrates that the fluorescence performance of N-CQDs was not significantly affected by pH during heavy metal ion detection, highlighting their strong practical applicability [50]. Consequently, a neutral pH (≈7) was used in subsequent fluorescence measurements for heavy metal ion detection.

### 2.2. Detection of Metal Ions by N-CQDs

#### 2.2.1. Selectivity Toward Metal Ions

**Figure 12:**
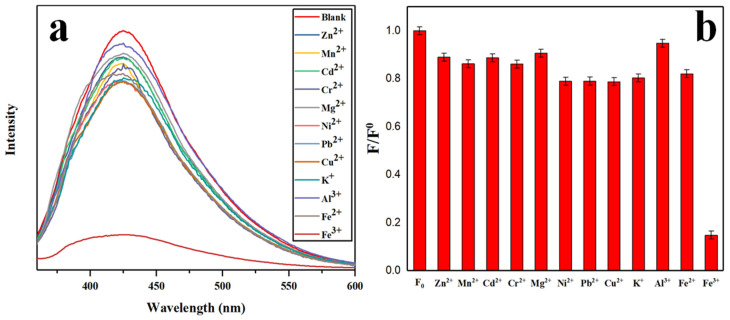
(**a**) Fluorescence spectra and (**b**) relative fluorescence intensity F/F_0_ of N-CQDs after adding different metal ions.

In order to investigate the selectivity of N-CQDs toward metal ions, twelve common metal ions—Zn^2+^, Pb^2+^, Ni^2+^, Mg^2+^, K^+^, Mn^2+^, Fe^3+^, Cu^2+^, Cd^2+^, Al^3+^, Fe^2+^, and Cr^2+^—were selected at a concentration of 2 mmol/L. It should be noted that Hg^2+^ was not included in the selectivity evaluation in this study, which represents a limitation of our work. Previous studies have shown that Hg^2+^ can also effectively quench CQD fluorescence through mechanisms similar to those of Fe^3+^, including electron transfer and coordination with surface functional groups [51]. A 1 mL aliquot of each ion solution was added to 4 mL of 10 mg/L N-CQDs solution, and the fluorescence intensity was measured after 2 min of reaction. Figure 12a shows the fluorescence spectra of different metal ions in N-CQD solution. As shown in Figure 12a, only Fe^3+^ induced a pronounced fluorescence quenching effect on N-CQDs, while the other eleven ions had negligible influence, demonstrating the strong selectivity of N-CQDs toward Fe^3+^ ions. The quenching mechanism may be attributed to the formation of non-fluorescent chelates between Fe^3+^ and oxygen- and nitrogen-containing functional groups on the N-CQDs surface. Due to the stronger complexation ability of Fe^3+^, the quenching effect is more significant than that of other ions [52]. The fluorescence intensity ratio (F/F_0_) of N-CQDs—where F_0_ and F represent the fluorescence intensity with and without metal ions, respectively, at 340 nm—is shown in Figure 12b. Although several other ions caused minor fluorescence changes (F/F_0_ ranging from 0.78 to 0.95), Fe^3+^ induced the most pronounced quenching effect (F/F_0_ = 0.14), which was significantly different from all other ions tested (*p* < 0.05, Duncan’s multiple range test).

#### 2.2.2. Linear Response Range for Fe^3+^ Detection

**Figure 13:**
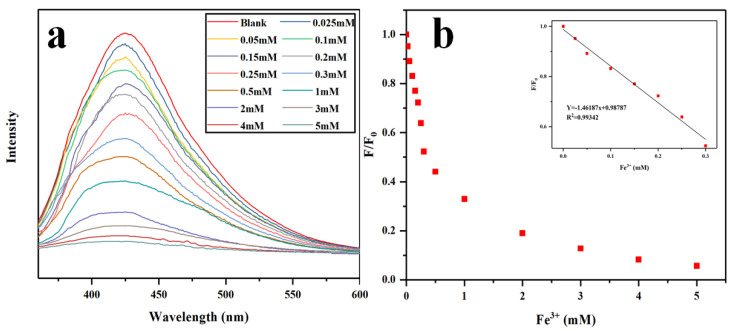
(**a**) Fluorescence spectra of N-CQDs solutions containing different concentrations of Fe^3+^. (**b**) Linear relationship between fluorescence intensity ratio (F/F_0_) and Fe^3+^ concentration (inset: linear regression of fluorescence response within the Fe^3+^ concentration range of 0 to 0.3 mmol/L).

To further explore the selective fluorescence quenching behavior of Fe^3+^ toward N-CQDs, a series of Fe^3+^ solutions with gradient concentrations (0–5 mmol/L) was prepared. Figure 13a presents the fluorescence spectra of N-CQDs solutions containing different Fe^3+^ concentrations. As shown in Figure 13a, the fluorescence spectra of N-CQDs exhibited a gradual decrease in intensity with increasing Fe^3+^ concentration, indicating a progressively stronger quenching effect. In order to determine the detection limit and response range of N-CQDs, the fluorescence intensity ratio (F/F_0_) was linearly fitted against the Fe^3+^ concentration. As illustrated in Figure 13b, a good linear relationship was observed within the concentration range of 0–0.3 mmol/L. At higher concentrations (up to 4 mmol/L), the fluorescence intensity continued to correlate linearly with Fe^3+^ concentration, and the quenching degree exceeded 90%. The inset of Figure 13b shows the linear regression equation Y = 0.98787 − 1.46187X within the Fe^3+^concentration range of 0 to 0.3 mmol/L, with a correlation coefficient (R^2^ = 0.99324). Based on the detection limit formula 3 ơ/m, the detection limit of N-CQDs for Fe^3+^ was calculated as 4.04 μmol/L, where σ represents the standard deviation of the blank N-CQDs and m is the slope of the regression line. This detection limit is considerably lower than the maximum allowable Fe^3+^ concentration (5.36 μmol/L) in drinking water according to the WHO Guidelines for Drinking-water Quality. These findings demonstrate that the synthesized N-CQDs exhibited high sensitivity and potential applicability for Fe^3+^ detection.

**Figure 14:**
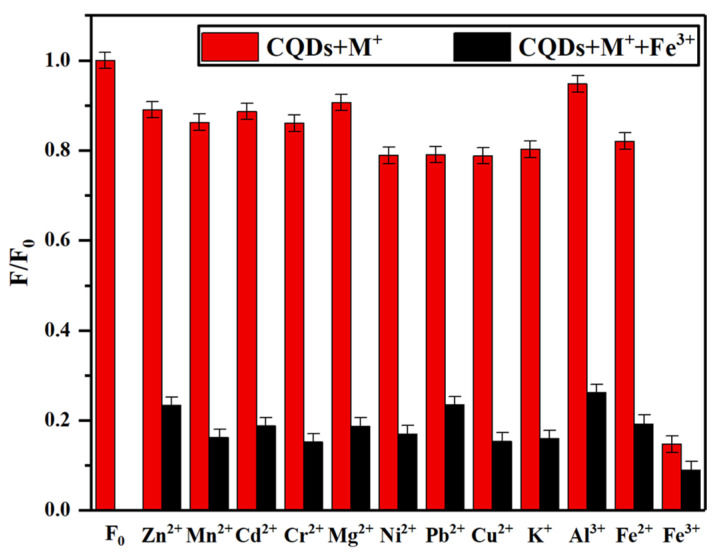
Relative fluorescence intensity of N-CQDs after addition of Fe^3+^ and various coexisting metal ions.

In real water environments, various ions and impurities are often present and may interfere with fluorescence-based detection. To evaluate the anti-interference performance of the synthesized N-CQDs in detecting Fe^3+^, the influence of eleven common metal ions coexisting with Fe^3+^ was investigated. The fluorescence intensity of the N-CQDs system was measured after adding equal concentrations and volumes of individual metal ion solutions and the Fe^3+^ solution. By comparing the fluorescence quenching of N-CQDs in the presence and absence of interfering ions, the anti-interference performance was assessed. As shown in Figure 14, the fluorescence intensity of N-CQDs significantly decreased in systems containing Fe^3+^, while other metal ions exhibited negligible effects. When Fe^3+^ coexists with other ions, the fluorescence quenching degree remains nearly identical to that of the N-CQDs–Fe^3+^ system alone, with the fluorescence intensity ratio remaining around 0.2. These findings indicate that the interference of other metal ions on Fe^3+^ detection is minimal, confirming that the prepared N-CQDs possess excellent selectivity and anti-interference capability for Fe^3+^ sensing.

### 2.3. Fluorescence Quenching Mechanism

The fluorescence property of carbon quantum dots refers to their ability to emit light when excited by an external light source. Upon illumination, the surface electrons of CQDs absorb photon energy and transition from the ground state to an excited state; as they return to the ground state, fluorescence emission occurs [53].

Fluorescence quenching refers to the interaction between fluorescent material molecules and quencher molecules, which mainly includes static quenching and dynamic quenching. The static quenching effect (SQE) occurs when the surface functional groups of CQDs interact with metal ions in the ground state to form a non-fluorescent “CQD-quencher” complex. Under excitation, this complex undergoes non-radiative transitions, resulting in fluorescence quenching. The dynamic quenching effect (DQE), on the other hand, results from collisions between excited-state CQDs and quenchers, during which energy transfer disrupts the electronic transition process, thereby reducing fluorescence intensity [54]. The fluorescence quenching of N-CQDs induced by Fe^3+^ is mainly a dynamic quenching mechanism. This is a characteristic of quenching induced by metal ions through electron transfer or energy transfer processes, and it follows the Stern–Volmer equation [55]:

$$\frac{F_{0}}{F}=1+K_{sv}\times Q$$

where F_0_ is the fluorescence intensity of N-CQDs without the quencher, F is the fluorescence intensity of N-CQDs after adding the quencher, K_sv_ is the fluorescence quenching constant, and Q represents the concentration of the quencher. The relationship between F_0_/F and Fe^3+^ concentration was analyzed to elucidate the fluorescence quenching mechanism of N-CQDs. Under purely static or dynamic quenching processes, the Stern–Volmer relationship is expected to yield a straight line. The Stem–Volmer equation obtained under both static and dynamic quenching becomes:

$$\frac{F_{0}}{F}=1+K_{sv}\times Q+K_{sv}\times Q^{2}$$

**Figure 15:**
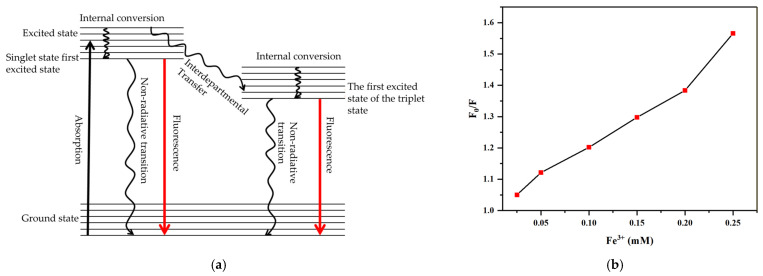
(**a**) Schematic illustration of the electron transition process N-CQDs; (**b**) fluorescence quenching efficiency of N-CQDs by Fe^3+^ within the concentration range of 0.025~0.25 mM.

As shown in Figure 15, a nonlinear relationship was observed between F_0_/F and the Fe^3+^ concentration in the range of 0.025~0.25 mmol/L, indicating that both static and dynamic quenching mechanisms are involved. Compared to other metal ions, Fe^3+^ exhibited a strong affinity for surface functional groups on N-CQDs (such as hydroxyl, carboxyl amino groups), forming non-luminous ground state complexes responsible for the static quenching effect [56]. Simultaneously, dynamic quenching occurs due to collision between excited-state N-CQDs and Fe^3+^ ions, facilitating non-radiative energy transfer and accelerating the return of electrons from the excited state to the ground state [57].

## 3. Conclusions

In this study, nitrogen-doped carbon quantum dots (N-CQDs) were successfully synthesized using chitin as a green and sustainable carbon source through a simple, environmentally friendly method without the need for external nitrogen doping. The obtained N-CQDs exhibited strong blue fluorescence under ultraviolet light. Comprehensive characterization revealed that the N-CQDs were spherical nanoparticles with an average diameter of approximately 2 nm and surfaces rich in hydrophilic functional groups such as carboxyl, hydroxyl and amino groups containing oxygen and nitrogen. The N-CQDs demonstrated excellent photostability over a wide pH range, a high carbonation yield of 54.46%, a fluorescence quantum yield of 34.33%, and strong photoluminescence, with optimal excitation and emission wavelengths at 340 nm and 425 nm, respectively. When applied to heavy metal ion detection, the N-CQDs exhibited a selective fluorescence quenching response toward Fe^3+^ ions, with no interference from other metal ions. Within the Fe^3+^ concentration range of 0–0.3 mmol/L, the fluorescence intensity of the N-CQDs showed a strong linear correlation with ion concentration, described by the regression equation Y = 0.98787 − 1.46187X (R^2^ = 0.99324), with a detection limit of 4.04 μmol/L. The quenching mechanism involved both dynamic and static effects. In summary, this study demonstrates a simple, green, and cost-effective approach for synthesizing N-CQDs and establishes their potential as a highly sensitive fluorescent probe for rapid Fe^3+^ detection in aqueous media, offering promising applications in environmental monitoring and water quality analysis.

## 4. Materials and Methods

### 4.1. Experimental Materials

The main materials and reagents utilized in this experiment are listed in Table 2. Deionized water was used throughout all tests. All reagents were of analytical grade and were used directly without further purification, unless otherwise specified.

### 4.2. Chitin Raw Material Purification

The commercial chitin had been pre-purified during industrial processing (protein content < 2%, and ash content < 2%, according to supplier specifications). To ensure complete removal of residual impurities and to prepare chitin suitable for nanogel synthesis, a secondary mild purification was performed as follows: Approximately 100 g of chitin was soaked in 2000 mL of 1 mol/L NaOH solution at room temperature for 12–16 h to achieve deproteinization. The sample was then filtered and rinsed with deionized water until the filtrate reached neutral pH. Subsequently, the sample was immersed in 2000 mL of 5% HCl solution at room temperature for 10–12 h to remove calcium carbonate (decarbonation), followed by thorough washing with deionized water. The alkaline and acid treatments were each repeated twice to ensure complete purification. After these treatments, the purified chitin was dispersed in 1000 mL of 0.3% NaClO_2_ buffer solution (pH = 4) and heated in an oil bath at 80 °C for 3 h for decolorization. The pH 4 buffer solution was prepared using citric acid and disodium hydrogen phosphate in a total volume of 20 mL, consisting of 7.71 mL of 0.2 mol/L disodium hydrogen phosphate and 12.29 mL of 0.1 mol/L citric acid. The product was filtered, washed repeatedly with deionized water until neutral, then dried and stored in a sealed container.

### 4.3. Preparation of Chitin Nanogel

Chitin nanogels were prepared via alkali/urea dissolution followed by high-speed shearing. Briefly, 1.5 g of purified chitin powder was dispersed in 100 g of pre-cooled (−4 °C) aqueous solution containing 8 wt% NaOH and 4 wt% urea. The mixture was then stored in an ultra-low temperature freezer at −30 °C for 4 h to freeze. Subsequently, the frozen mixture was removed and allowed to thaw at room temperature under magnetic stirring at 200 rpm. Each thawing step lasted approximately 30–45 min, until the mixture returned to a liquid state. This freeze–thaw cycle (freezing at −30 °C for 6 h followed by thawing at room temperature with stirring) was repeated 3 times to promote complete dissolution of chitin through disruption of hydrogen bonding networks.

The resulting chitin solution was then homogenized using a high-speed disperser (T 18 Digital ULTRA-TURRAX, IKA, Staufen, Germany) at 10,000 rpm for 25 min in an ice-water bath to prevent overheating. During homogenization, the dissolved chitin chains reassembled into nano-sized gel particles due to reconstruction of hydrogen bonding networks under mechanical shearing. The obtained nanogel dispersion appeared translucent and was stable. The dispersion was centrifuged at 8000 rpm for 10 min to remove any large aggregates, and the supernatant was collected. The nanogels were then purified by repeated washing cycles: centrifugation (8000 rpm, 10 min), removal of supernatant, and redispersion in deionized water. This washing process was repeated 5–6 times until the pH reached neutrality (pH 7.0). Finally, the purified CNGs were dialyzed against deionized water using a cellulose dialysis membrane for 5 days, with water changes refreshed every 12 h, to completely remove residual NaOH and urea. The purified CNGs dispersion was stored at 4 °C until further use.

### 4.4. Surface Modification of Chitin Nanogel

#### 4.4.1. Preparation of Amino-Chitin Nanogels (DE-CNGs)

Amino-chitin nanogels were prepared by alkaline deacetylation of chitin nanogels under varying reaction durations. Specifically, 30 wt% NaOH was added to 100 g of chitin nanogels, and the mixture was heated in an oil bath at 90 °C for 1, 2, and 3 h, respectively. After cooling to room temperature, the product was centrifuged once and subsequently dialyzed against deionized water until a neutral pH was achieved. This process yielded amino-chitin nanogels (DE-CNGs) with different surface positive charge densities, depending on the deacetylation time.

#### 4.4.2. Preparation of Carboxylated Chitin Nanogels (CO-CNGs)

Carboxylated chitin nanogels were synthesized through a TEMPO-mediated oxidation of chitin nanogels using varying amounts of NaClO. Briefly, 0.1 g NaBr and 0.016 g Tempo reagent were added to 100 g of 1% chitin nanogel dispersion. The reaction pH was continuously monitored using a PHS-3C pH meter (Remco Instruments, Shanghai, China) and maintained under magnetic stirring. Subsequently, 3 mmol, 6 mmol and 9 mmol NaClO were added dropwise, ensuring that an additional drop was introduced each time the pH decreased to about 9.5. During the oxidation process, 0.5 mol/L NaOH solution was added dropwise to maintain the pH around 10, until NaOH consumption ceased (indicated by a pH change in less than 0.4 within 5 min). The reaction was then terminated by adding a few drops of ethanol. The resulting carboxylated chitin nanogels (CO-CNGs), possessing different surface negative charge densities, were purified by dialysis against deionized water until neutrality was achieved.

### 4.5. Hydrothermal Synthesis of N-CQDs from Chitin Nanogels

The N-CQDs were synthesized via hydrothermal carbonization of the prepared nanogels (DE-CNGs or CO-CNGs), recorded as amino-N-CQDs and carboxyl-N-CQDs. Specifically, 10 mL of nanogel dispersion (solid content: 1 wt%) was transferred into a 25 mL Teflon-lined stainless-steel autoclave. The sealed autoclave was then heated in an oven at 200 °C for 6 h. After the reaction, the autoclave was cooled to room temperature naturally. The resulting brown suspension was centrifuged at 10,000 rpm for 15 min to remove large aggregates. The supernatant containing N-CQDs was collected and dialyzed against deionized water for 48 h using a dialysis membrane, with water refreshed every 12 h, to remove small molecular impurities. The purified N-CQDs dispersion was stored at 4 °C for subsequent characterization and application.

### 4.6. Characterization

The microstructure of the prepared nanogels (DE-CNGs or CO-CNGs) and CQDs were examined using transmission electron microscopy (TEM, FEI Tecnai G2 Spirit Bio TWIN, Hillsboro, OR, USA) at an accelerating voltage of 200 kV. Samples were prepared by dropping the samples dispersion onto copper grids and allowing them to dry naturally at room temperature. The nanogels (DE-CNGs or CO-CNGs) were also examined via field emission scanning electron microscopy (FE-SEM, ZEISS, Oberkochen, Germany) after gold coating. The zeta potential and particle size distribution of the nanogels were determined using a Nano ZS90 potential-particle size analyzer (Malvern Panalytical, Westborough, Westborough, MA, USA). The surface functional groups of the nanogels and N-CQDs were analyzed by a Fourier transform infrared (FTIR) spectrometer (Nicolet 5700, Thermo Nicolet, Waltham, MA, USA). Freeze-dried samples were finely ground, mixed with potassium bromide, and pressed into pellets. The spectra were recorded in the 4000~500 cm^−1^ range. The phase structure information of activators was studied by a Horiba Scientific LabRAM HR Evolution Raman spectroscopy system with a 532 nm excitation laser. X-ray photoelectron spectroscopy (XPS) measurements were performed on a Thermo Scientific K-Alpha X-ray photoelectron spectroscope (Thermo Fisher Scientific, East Grinstead, UK) with Al Kα radiation. The optical absorption properties were characterized using an ultraviolet-visible spectrophotometer (TU-1901, Beijing General Instrument, Beijing, China) with a medium scanning speed over a 200~900 nm wavelength range. Fluorescence properties were measured using a fluorescence spectrophotometer (Fluorolog-3, Horiba Jobin Yvon, Edison, NJ, USA) after preheating the instrument for 30 min prior to analysis.

The fluorescence quantum yield (QY) of the N-CQDs was determined using a relative reference method. The fluorescence quantum yield represents the efficiency of photon utilization in a photochemical process, defined as the ratio of the number of photons emitted by a fluorescent material to the number of photons absorbed. Quinine sulfate, dissolved in 0.1 mol/L sulfuric acid, was used as the reference standard, with a reported QY of 54% under 350 nm excitation. The fluorescence intensity and absorbance of both the reference and the N-CQD samples were measured, and the relative fluorescence quantum yield was calculated accordingly. To investigate the effect of pH on the fluorescence properties of N-CQDs, the pH of the diluted CQDs solution was adjusted using hydrochloric acid and sodium hydroxide to obtain a gradient ranging from pH 0 to 14. The fluorescence intensity was measured at the optimal excitation wavelength, ensuring consistent CQD concentration across samples. Each sample was measured five times, and the average value was recorded as the final result.

### 4.7. Selectivity and Anti-Interference Experiments of N-CQDs

In order to evaluate the selectivity of N-CQDs toward various metal ions, 12 common metal ion salts—Zn^2+^, Pb^2+^, Ni^2+^, Mg^2+^, K^+^, Mn^2+^, Fe^3+^, Cu^2+^, Cd^2+^, Al^3+^, Fe^2+^, and Cr^2+^—were each prepared as 2 mmol/L aqueous solutions. Subsequently, 12 quartz cuvettes were filled with 4 mL of 10 mg/L N-CQDs solution, followed by the addition of 1 mL of each metal ion solution. After thorough mixing and standing at room temperature for 1 min, the fluorescence spectra were recorded to assess ion-specific responses. To determine the detection range of N-CQDs for Fe^3+^, Fe^3+^ solutions with concentrations of 0 (blank), 0.025, 0.05, 0.1, 0.15, 0.2, 0.25, 0.3, 0.5, 1, 2, 3, 4, and 5 mmol/L were prepared by appropriate dilution. A total of 14 quartz cuvettes were each filled with 4 mL of 10 mg/L N-CQDs solution, followed by the addition of 1 mL of Fe^3+^ solution at the designated concentration (or 1 mL deionized water for the blank). After thorough mixing and being left for 1 min at room temperature, the fluorescence spectra were measured to establish the relationship between N-CQDs and Fe^3+^ concentration. In order to further examine the anti-interference capability of N-CQDs for Fe^3+^ detection, 12 quartz cuvettes were again prepared with 4 mL of 10 mg/L N-CQDs solution. Then, 1 mL of each 2 mmol/L metal ion solution was added, thoroughly mixed, followed by the addition of an equal volume (1 mL) of 2 mmol/L Fe^3+^ solution. After mixing and standing for 1 min at room temperature, the fluorescence spectra were recorded. All experiments were repeated five times, and the average values were reported as the final data to ensure reliability and reproducibility.

The fluorescence quantum yield (QY) was determined using the relative slope method with quinine sulfate in 0.1 M H_2_SO_4_ (QY = 54%) as the reference standard. Five concentrations of both quinine sulfate (0.005, 0.010, 0.015, 0.020, 0.025 mg/mL) and N-CQDs (2, 4, 6, 8, 10 mg/L) were prepared to ensure absorbance values below 0.1 at 340 nm (excitation wavelength). The integrated fluorescence intensity (IF) from 380 to 600 nm was plotted against absorbance (A) for each series. The QY of N-CQDs was calculated using:

$$QY_{\mathrm{CQDs}}\;=\;QY_{\mathrm{QS}}\;\times \;\left(\frac{{\mathrm{Slope}}_{\mathrm{CQDs}}}{{\mathrm{Slope}}_{\mathrm{QS}}}\right)\;\times \;\left(\frac{\eta_{\mathrm{CQDs}}^{2}}{\eta_{\mathrm{QS}}^{2}}\right)$$

where η is the refractive index of the solvent (water: 1.333; 0.1 M H_2_SO_4_: 1.333). The previously mentioned concentration of 10 mg/L represented the highest concentration used in the dilution series, ensuring measurements remained in the linear low-absorbance regime.

For fluorescence intensity measurements, all N-CQD samples were diluted to 10 mg/L in deionized water. Fluorescence spectra were recorded using an excitation wavelength of 340 nm, with emission collected from 380 to 600 nm. Excitation and emission slit widths were both set to 5 nm, and measurements were conducted at room temperature (25 ± 1 °C).

### 4.8. Statistical Analysis

Statistical analyses were performed using analysis of variance (ANOVA) with SPSS software version 24.0. Duncan’s multiple range test was applied for pairwise comparisons, with significance set at *p* < 0.05.

## Figures and Tables

**Table 1:** Zeta Potentials of DE-CNGs and CO-CNGs under different conditions.

Reaction Conditions	Potential (mV)
DE–CNGs (1 h)	27.2
DE–CNGs (2 h)	29.4
DE–CNGs (3 h)	34.3
CO–CNGs (3 mmol)	−12.1
CO–CNGs (6 mmol)	−15.4
CO–CNGs (9 mmol)	−22.2

**Table 2:** Main experimental materials and reagents.

Reagent Name	Specification	Manufacturer
Chitin	Commercial grade Purity ≥ 85%, Deacetylation degree < 5%	Zhejiang Golden Shell Co., Ltd. (Taizhou, China)
NaOH	AR	Sinopharm Group Chemical Reagent Co., Ltd. (Shanghai, China)
Urea	AR	Sinopharm Group Chemical Reagent Co., Ltd. (Shanghai, China)
HCl	35%	Sinopharm Group Chemical Reagent Co., Ltd. (Shanghai, China)
Citric acid	AR	Sinopharm Group Chemical Reagent Co., Ltd. (Shanghai, China)
Disodium hydrogen phosphate	AR	Sinopharm Group Chemical Reagent Co., Ltd. (Shanghai, China)
Sodium bromide	AR	Zhengzhou Pini Chemical Reagent Factory (Zhengzhou, China)
TEMPO	98%	Zhengzhou Pini Chemical Reagent Factory (Zhengzhou, China)
Potassium bromide	SP	Shanghai Aladdin Biochemical Technology Co., Ltd. (Shanghai, China)
Manganese Sulfate monohydrate	AR	Sinopharm Group Chemical Reagent Co., Ltd. (Shanghai, China)
Lead chloride	AR	Tianjin Kemi Ou Chemical Reagent Co., Ltd. (Tianjin, China)
Magnesium sulfate heptahydrate	AR	Sinopharm Group Chemical Reagent Co., Ltd. (Shanghai, China)
Ferric chloride hexahydrate	AR	Sinopharm Group Chemical Reagent Co., Ltd. (Shanghai, China)
Zinc Sulfate heptahydrate	AR	Sinopharm Group Chemical Reagent Co., Ltd. (Shanghai, China)
Chromium chloride	AR	Tianjin Kemi Ou Chemical Reagent Co., Ltd. (Tianjin, China)
Potassium chloride	AR	Sinopharm Group Chemical Reagent Co., Ltd. (Shanghai, China)
Nickel chloride	99%	Shanghai Aladdin Biochemical Technology Co., Ltd. (Shanghai, China)
Copper sulfate pentahydrate	AR	Tianjin Kemi Ou Chemical Reagent Co., Ltd. (Tianjin, China)
Ferrous sulfate heptahydrate	AR	Sinopharm Group Chemical Reagent Co., Ltd. (Shanghai, China)
Quinine sulfate	98%	Shanghai Aladdin Biochemical Technology Co., Ltd. (Shanghai, China)

## Data Availability

The original contributions presented in this study are included in the article. Further inquiries can be directed to the corresponding authors.
